# Using the Infrastructure of State Aging Services to Promote Prevention Behavior

**DOI:** 10.5888/pcd15.170567

**Published:** 2018-07-05

**Authors:** Steven M. Albert, Jennifer King, Jennifer R. Jones, Michelle E. Danielson, Yuae Park, Anne B. Newman

**Affiliations:** 1 Department of Behavioral and Community Health Sciences, University of Pittsburgh, Pittsburgh, Pennsylvania; 2 Clinical Translational Science Center, University of Pittsburgh, Pittsburgh, Pennsylvania; 3 Department of Epidemiology, University of Pittsburgh, Pittsburgh, Pennsylvania

## Abstract

**Introduction:**

State infrastructure for aging services, such as programs in county senior centers, can help promote prevention of chronic disease and reach large numbers of older adults. The objective of our study was to assess how well such infrastructure can support prevention efforts.

**Methods:**

The University of Pittsburgh CDC Prevention Research Center partnered with the Pennsylvania Department of Aging APPRISE program to deliver the 10 Keys to Healthy Aging program. APPRISE is a Medicare counseling program offered at senior centers; the 10 Keys is a series of behavior-activation workshops for people aged 50 or older that cover recommendations of the US Preventive Services Task Force and other evidence-based recommendations for health promotion. We assessed implementation, increases in prevention knowledge, and maintenance of prevention behavior.

**Results:**

From 2013 through 2016, 1,534 adults at 83 sites participated in the program; 1,044 (68.1%) completed at least 8 of 10 Keys workshops. A total of 736 adults (mean [standard deviation] age, 74.9 [8.3] y) voluntarily completed a 14-item pretest and posttest of prevention knowledge; respondents’ knowledge score increased from 61.5% to 78.5% correct (*P* < .001). In a subsample (n = 339) reporting on their own prevention behaviors at baseline, quiz scores and prevention behaviors were correlated (*r* = 0.30, *P* < .001). In monthly telephone follow-up with 147 respondents over 6 months, maintenance of prevention behaviors was strong in the areas of physical activity and hypertension management and significantly higher for people completing a greater number of Keys workshops.

**Conclusion:**

Prevention behavior can be activated in aging services settings and can be incorporated into daily routines.

## Introduction

Healthy aging requires “adoption and maintenance of attitudes and behaviors known to promote health and wellbeing, and the effective use of health services to prevent or minimize the impact of acute and chronic disease on function” (1,2). Empowering older adults to use preventive services to reduce the risk of chronic disease in later life is central to this effort (3). A growing number of evidence-based programs focus on particular health risks (such as falls or sedentary behavior), disabling diseases (such as diabetes or arthritis), and management of chronic conditions (such as chronic pain and medication management).

Less common are programs designed to activate prevention behavior in the setting of older adults’ contacts with health care professionals. Although physician practice-based interventions have boosted some prevention efforts, such as vaccination (4), many opportunities for prevention in this setting are missed. The introduction of the annual Medicare Wellness Visit as a covered benefit was designed to address this underinvestment in prevention in primary care, but the benefit is underutilized, with only 15% of Medicare-eligible adults taking advantage of it (5). A program that alerts older people to prevention guidelines, gives people tools for incorporating prevention into daily behavior, and prepares them to talk to physicians about prevention could boost adherence to guidelines of the US Preventive Services Task Force and related guidelines and lower chronic disease risk.

Determining whether such a program could be delivered within the infrastructure of existing state aging services would be valuable. State aging services include senior centers in counties that already attract older people for services and recreation, as well as staff and volunteers to deliver programs, track attendance, and record outcomes. Use of state aging services infrastructure may be a promising way to deliver prevention to older adults on a large scale.

The objective of our study was to assess how well such infrastructure can support prevention efforts. To that end, the University of Pittsburgh CDC [Centers for Disease Control and Prevention] Prevention Research Center partnered with the Pennsylvania Department of Aging APPRISE program to deliver the 10 Keys to Healthy Aging program. APPRISE is a Medicare counseling program offered at senior centers as part of the state health insurance program; the 10 Keys program is a series of behavior-activation workshops for people aged 50 or older that cover US Preventive Services Task Force guidelines and other evidence-based recommendations for health promotion. We assessed increases in prevention knowledge and maintenance of prevention behavior among participants in these workshops. We sought to answer the following questions: Will older adults attend a health promotion program offered in a Medicare–senior center–workshop setting without direct researcher involvement? Will participants significantly increase knowledge of prevention behaviors? Is level of prevention knowledge associated with prevention behaviors? And finally, do people incorporate prevention behavior into daily routines after completing the program?

## Methods

### 10 Keys to Healthy Aging

The 10 Keys to Healthy Aging (6) is a behavioral-activation program designed to empower adults aged 50 or older to seek prevention in health care encounters (7). The 10 Keys participant workbook (in English [8] and Spanish [9]) and instructor manual are available electronically from the CDC Prevention Research Center at the Center for Aging and Population Health, University of Pittsburgh (www.caph.pitt.edu). Internet-based training is available for instructors, who are certified by completing online quizzes. To date, more than 500 instructors have been certified.

Briefly, the 10 Keys program targets the most common chronic health conditions that contribute to preventable disability, their risk factors (which are often shared across conditions), and prevention strategies. The targeted medical conditions include coronary heart disease and stroke, breast and colon cancer, pneumonia and chronic lung disease, musculoskeletal health (osteoporosis and sarcopenia), and depression. The 10 Keys are risk factor prevention strategies and include the following: 1) blood pressure control (systolic blood pressure <120 mm Hg), 2) cholesterol control (low-density lipoprotein [LDL] <100 mg/dL), 3) glucose control (<100 mg/dL), 4) smoking cessation, 5) influenza immunization (past year) and pneumonia immunization (ever), 6) breast and colon cancer screening, 7) osteoporosis screening and muscle-strengthening exercises, 8) physical activity (moderate activity >2.5 hours per week), 9) social contact at least once per week, and 10) awareness and treatment of depression symptoms.

The 10 Keys program provides strategies for behavior change that address the 10 prevention targets. For example, for blood pressure, participants handle a blood pressure cuff, measure their own blood pressure, and determine whether they need to take action to lower it. They identify steps that will lower blood pressure, including knowing their blood pressure values, reducing sodium in diets, and understanding the role of medication in controlling hypertension. For example, participants identify sodium content in foods by reviewing labels and reporting back to other participants 4 food items that they discovered are high in sodium. The program emphasizes activating behavior change through hands-on efforts in a group setting, guided by the instructor. Participants complete 2-hour workshops once per week for 10 weeks or twice per week for 5 weeks.

### Medicare counseling program, Pennsylvania APPRISE

Pennsylvania APPRISE is a free health insurance counseling program that provides information about Medicare, Medicare supplemental insurance, Medicaid, and long-term care insurance. Medicare counseling is a component of State Health Insurance Assistance Programs (SHIP), a federal program to provide counseling and assistance to people with Medicare and their families. Beginning in 2013, the Pennsylvania Department of Aging received funding through the Medicare Improvement for Patients and Providers Act of 2008 to link the APPRISE counseling program and the 10 Keys program (http://www.aging.pa.gov/aging-services/health-wellness/Pages/10-Keys-to-Healthy-Aging.aspx).

In each workshop, APPRISE presentations on Medicare benefits and relevant Keys were paired. For example, the smoking cessation Key was paired with Medicare tobacco cessation counseling. The cancer screening Key was paired with Medicare coverage of mammograms, Papanicolaou (Pap) tests, and prostate and colorectal cancer screening. The immunization Key was paired with Medicare coverage of influenza, pneumococcal, and hepatitis B immunization. The blood glucose Key was paired with diabetes screening and self-management training, and the LDL cholesterol Key with cardiovascular disease screening. The other Keys were handled similarly. A certified 10 Keys workshop leader and APPRISE counselor were paired at each site.

Regional APPRISE coordinators supervised the on-site programs in each county. All 10 Keys workshop leaders completed web training and were certified after completing quizzes for each Key. The research team had contact with workshop leaders only through teleconferences, which took place at the launch of the program each year and at periodic check-ins, and had no contact with workshop participants. However, the principal investigator (A.B.N.) and a nurse were available to answer questions by email and by toll-free number, if workshop leaders had questions.

### Sample

Participants in 10 Keys–APPRISE were invited from among people who regularly attended senior center events and people attending community health and wellness events. Flyers announcing the program were displayed at the local Area Agency on Aging, senior centers, and other venues, as well as announced in monthly senior community newsletters, newspapers, and public service announcements.

Participants in the program were not required to complete questionnaires or answer questions beyond the Pennsylvania Department of Aging’s enrollment requirements. As a quality assurance effort, participants were invited to complete a prevention knowledge quiz anonymously at the start and end of the program, in which they also provided basic demographic information (sex, age, race [white, black, Asian, other], and ethnicity [Hispanic, non-Hispanic]). They could also complete, again anonymously, a short questionnaire on current prevention behavior. We were able to match pretest and posttest quizzes and prevention behavior reports because sites generated their own identifiers for participants. The research team did not have access to personal identifiers.

Toward the end of the project, we invited participants to sign a consent form for telephone follow-up to assess incorporation of prevention behavior into daily routines. This component of the research was reviewed and approved by the institutional review board of the University of Pittsburgh.

### Measures

The 14-item prevention knowledge quiz (Appendix) was designed to assess awareness of chronic disease prevention guidelines. We did not score the social interaction question, and because of a change in mammography guidelines we accepted 1 or 2 years as a correct answer to how often women aged 50 to 74 in the general population should have a mammogram. Workshop leaders collected forms before the first workshop and during the last, and forms were sent to the research team as programs finished.

The prevention behavior inventory asked participants if they were currently practicing up to 9 different prevention behaviors. Participants completed the inventory at the start of the program. These roughly matched the 10 Keys and included currently not smoking, influenza immunization in the previous 12 months, ever had pneumonia immunization, knew their systolic blood pressure value, ever had colon cancer screening, ever had bone-density screening, knew their blood glucose value, exercised 2.5 or more hours per week, and knew their LDL cholesterol value. We were not able to validate these self-reports and considered the ability to give a value as a sign of prevention behavior.

The telephone follow-up involved brief monthly calls to ascertain the presence and frequency of prevention behaviors. We asked questions about 3 broad domains of prevention during the previous 30 days over multiple months. All participants were asked about physical activity: whether they exercised at least 2.5 or more hours per week and engaged in weekly muscle-strengthening exercises. Participants reporting hypertension were asked if they had checked their blood pressure and limited salt in their diet. Finally, people reporting contact with a health care professional were asked if they were able to ask questions important to them to tap greater empowerment in contact with physicians and other providers.

### Analyses

To assess uptake and dissemination of the program, we tabulated the number of sites, counties, and participants in the program between its initial rollout in 2013 and last year of full data collection, 2016. From site workshop counts of attendance, we noted the total number of participants and the total number completing 8 or more of the 10 Keys–APPRISE workshops.

To compare pretest and posttest quiz performance, we calculated the proportion answering each question correctly at each time point and computed the total percentage correct. We used the paired-sample McNemar test to determine whether the proportion correct for each posttest question and total score differed significantly from the pretest score. We also determined whether baseline performance on the prevention knowledge quiz and reports of prevention behavior were correlated.

Finally, because we had no control condition, we compared reports of prevention behavior among people not fully engaged in the program (<5 Keys completed) versus a more engaged group (≥5 Keys completed). People in this subanalysis received multiple monthly follow-up telephone calls, allowing us to calculate the proportion of months over follow-up in which people in the 2 groups reported the behavior and to compare proportions across the 2 groups using independent samples *t* tests.

## Results

During the study period, 1,534 people attended at least one workshop and 1,044 (68.1%) completed 8 or more Keys workshops (Table 1). The program grew from 93 participants in its pilot year in 2013 to 694 participants in 2016. Eleven to 14 of Pennsylvania’s 57 Area Agencies on Aging (AAA), offer the program each year; these participating AAAs represented mostly rural counties.

**Table 1:** Program Uptake: Sites and Participation in 10 Keys–APPRISE Program^a^, by Year, Pennsylvania, 2013–2016

Factor	2013	2014	2015	2016	Total
No. of sites that hosted program^b^	7	15	23	38	83
No of participating counties or Area Agency on Aging regions	5	12	14	11	14
No. of people who attended ≥1 workshop^c^	93	217	530	694	1,534
No. (%) of participants who completed ≥8 workshops^c^	60 (64.5)	129 (59.5)	391 (73.7)	464 (66.9)	1,044 (68.1)

a 10 Keys to Healthy Aging–APPRISE is a Medicare counseling program consisting of a series of behavior-activation workshops for people aged 50 or older that cover recommendations of the US Preventive Services Task Force and other evidence-based recommendations for health promotion.

b 80 nonduplicated sites hosted programs.

c Participants complete 2-hour workshops once per week for 10 weeks or twice per week for 5 weeks.

Of the 1,534 participants, 1,085 (70.7%) completed the pretest prevention knowledge quiz (Figure); 736 completed both the pretest and posttest and could be matched by site-assigned identifiers. The mean (standard deviation [SD]) age of participants completing the pretest was 75.4 (8.5). Mean (SD) age in the sample completing both pretests and posttests was 74.6 (9.9). The 2 sets of participants did not differ in proportion female (84.1%) or white (89.8%). Of the 736 participants who completed pretests and posttests, 339 provided responses at baseline to survey questions about their own prevention behavior. The mean (SD) age in this subsample was 75.6 (8.3) and also did not differ by sex or race distribution from the other samples.

**Figure:**
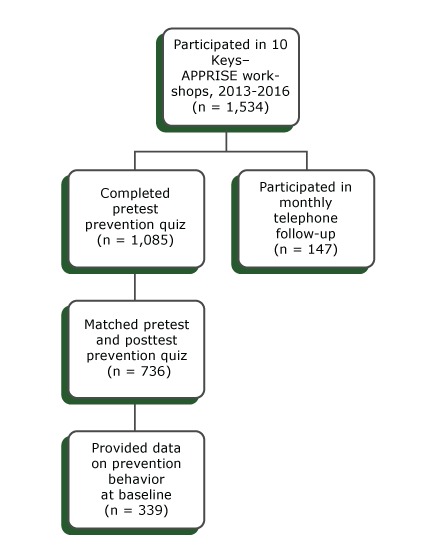
Participants in the 10 Keys to Healthy Aging–APPRISE program, Pennsylvania, 2013–2016. The program is a Medicare counseling program consisting of a series of behavior-activation workshops for people aged 50 or older that cover recommendations of the US Preventive Services Task Force and other evidence-based recommendations for health promotion.

### Performance on prevention knowledge quiz

Scores in the 13-item prevention knowledge quiz increased from a mean (SD) of 8.0 (2.3), 61.5% correct, at the beginning of the program, to 10.2 (2.4), 78.5% correct (*P* < .001), at its end. The proportion correct for each knowledge item increased significantly except for the items on smoking, bone loss, and osteoarthritis, in which over 90% of participants had correct answers at pretest.

Older age was associated with lower scores on the quiz, but the effect was small (Pearson *r* = −0.12 for pretest [*P* = .001], −0.08 posttest [*P* = .03]). Men performed more poorly than women on both the pretest (7.3 vs 8.1 correct, *P* < .001) and posttest (9.6 vs 10.3, *P* = .03). Nonwhite participants performed more poorly than white participants on the pretest (6.9 vs 8.1, *P* < .001), but the race groups did not differ at posttest (10.3 vs 10.2), suggesting greater uptake of prevention knowledge among racial minority participants.

### Relationship of prevention knowledge to self-reported prevention behavior

The 339 participants who provided information on their prevention behavior reported a mean (SD) of 5.8 (1.8) of 9 behaviors surveyed (Table 2). These ranged from a low of 29.8% for knowing one’s LDL cholesterol value to a high of 95.6% for not smoking in the previous 12 months. Age was not significantly correlated with number of prevention behaviors. Women reported more prevention behaviors than men (6.0 vs 5.0, *P* = .01) and white participants reported more than nonwhite participants (5.9 vs 5.0, *P* = .003). Prevention quiz scores and baseline prevention behaviors were correlated (Pearson *r* = 0.30, *P* < .001).

**Table 2:** Prevention Behaviors at Baseline, Participants (n = 339^a^) in 10 Keys–APPRISE Program^b^, Pennsylvania, 2013–2016

Prevention Behavior	No. (%)^c^
Does not smoke	324 (95.6)
Received influenza immunization in previous 12 months	272 (80.2)
Ever received pneumonia immunization	258 (76.1)
Knows own systolic blood pressure value	254 (74.9)
Ever been screened for colon cancer	242 (73.3)
Ever had bone-density screening	233 (68.7)
Knows own blood glucose value	162 (47.8)
Exercises ≥2.5 hours per week	126 (38.7)
Able to give low-density lipoprotein cholesterol value	101 (29.8)
Mean (SD) no. of prevention behaviors	5.8 (1.8)

a Of 1,534 people who attended a workshop in 2013–2016, 1,085 completed a pretest prevention quiz. We matched posttest quizzes for 736 of these participants. Of these 736 participants, 339 completed a questionnaire at baseline on their own prevention behavior.

b 10 Keys to Healthy Aging–APPRISE is a Medicare counseling program consisting of a series of behavior-activation workshops for people aged 50 or older that cover recommendations of the US Preventive Services Task Force and other evidence-based recommendations for health promotion.

c All values are percentages, unless otherwise indicated. Analyses limited to people providing information about prevention behavior at baseline (n = 326–339).

### Incorporation of prevention knowledge into daily behavior

During the study period, 147 participants participated in monthly telephone follow-up and completed 662 monthly assessments; 103 (70.1%) had 3 or more months of follow-up. The mean age (SD) of this subsample was 72.7 (8.1). Of the 147 participants followed, 122 (83.2%) were women, 43 (29.3%) reported attending college, and 134 (91.2%) reported contact with a health care professional during follow-up. At the start of follow-up, 105 (71.4%) reported hypertension.

Participants completing fewer than five 10 Keys workshops (n = 55) were less likely to incorporate prevention behavior into daily routines than people completing 5 or more Keys workshops (n = 88) (Table 3 [4 were missing information on number of workshops attended]). Participants completing 5 or more workshops reported doing weekly muscle-strengthening exercises for a greater proportion of months of follow-up than did participants completing fewer than 5 workshops (76.0% vs 63.0% of months of follow-up, *P* = .048). Participants completing 5 or more workshops also reported a greater proportion of months in which they checked blood pressure (84.1% vs 72.6%, *P* = .059).

**Table 3:** Maintenance Prevention Behaviors of Program Participants at Follow-Up^a^ (n = 143), 10 Keys–APPRISE Program^b^, Pennsylvania, 2013–2016

Domain	Proportion of Follow-Up Months in Which Participants Reported Behavior		Difference in Proportions, Independent Samples *t* Test Statistic (*P* Value)
	Among Participants Who Completed <5 Key Workshops (n = 55)	Among Participants Who Completed ≥5 Key Workshops (n = 88)	
**Physical activity (asked of all participants [n = 147])**			
Exercises ≥2.5 hours per week	87.8	91.2	0.84 (.40)
Engage in weekly muscle-strengthening exercises	63.0	76.0	2.07 (.048)
**Empowerment in communicating with health care providers (asked of participants reporting visit to health care professional [n = 134])**			
Is able to ask questions that are important to him or her	50.7	43.2	1.25 (.22)
**Hypertension (asked of participants reporting hypertension at baseline [n = 105])**			
Checks blood pressure	72.6	84.1	1.92 (.059)
Limits salt in diet	87.1	95.8	1.65 (.11)

a All participants were invited to take part in follow-up to assess incorporation of prevention behavior into daily routines; 147 people elected to participate (data were missing for 4 participants), and 662 assessments were completed. Follow-up consisted of monthly telephone calls during 6 months after the final 10 Keys workshop; 103 participants (70.1%) had ≥3 months follow-up.

b 10 Keys to Healthy Aging–APPRISE is a Medicare counseling program consisting of a series of behavior-activation workshops for people aged 50 or older that cover recommendations of the US Preventive Services Task Force and other evidence-based recommendations for health promotion.

## Discussion

The 10 Keys–APPRISE collaboration shows that prevention efforts for older adults can be delivered at scale by using existing aging-services infrastructure. We augmented a Medicare plan counseling program with chronic disease prevention workshops. No additional staff members were required, because the 10 Keys–APPRISE workshops were offered by current staff or volunteers. Training in the 10 Keys, however, was required, which we made available through internet-based certification and which was reinforced by Pennsylvania Department of Aging teleconferences. The APPRISE program ensured that Medicare counselors were trained. Small Medicare Improvement for Patients and Providers Act grants were sufficient to boost publicity and make 10 Keys workbooks available. In short, the program used mostly existing resources during 4 years to reach more than 1,500 people. Without on-site involvement of the research team, nearly 70% of participants completed at least 8 of the 10 workshops.

The program was effective, increasing correct answers on the prevention knowledge quiz from 61.5% to 78.5%. Comparing performance on the quiz to self-report of prevention behaviors suggests that greater understanding of prevention targets is related to prevention behavior.

Explicitly pairing 10 Keys prevention targets with Medicare services may be an effective way to boost prevention behavior, because reaching prevention targets often involves collaboration with health care professionals. Our exploratory analysis showed that greater engagement in the 10 Keys–APPRISE program was associated with greater likelihood of incorporating prevention behaviors into daily routines. Although not a replacement for a randomized trial, this study suggests that greater behavioral activation to support prevention may increase adoption of prevention behaviors.

It may be helpful to compare the 10 Keys–APPRISE program with the Chronic Disease Self-Management Program (CDSMP). CDSMP was originally assessed in a randomized controlled trial (10,11) and more recently in a large national demonstration (12–14). CDSMP is designed for people with at least one chronic condition and emphasizes strategies to mitigate disease sequelae (fatigue, pain, frustration, and isolation). The CDSMP effort shows that elements of prevention can be taught in the setting of community-based disease management workshops.

The 10 Keys–APPRISE program differs from CDSMP in that it emphasizes taking advantage of preventive services to promote wellness and reduce the risk of chronic conditions. The link to Medicare clinical preventive services makes this connection explicit. Thus, an important outcome for future evaluation of the 10 Keys program is to determine whether older adults increase their use of the Medicare Wellness Visit and clinical prevention services.

An alternative approach is to build risk factor reduction and prevention efforts directly into clinical encounters. This strategy was adopted in the My Own Health Report (MOHR) study, in which patients completed a health risk appraisal before their clinical encounters. After reviewing the health risk appraisal, clinicians worked with patients to develop strategies to reduce these risks and reach prevention goals (15,16). In this randomized trial, physicians in intervention practices were more likely to ask about risk factors and work with patients to set goals for risk reduction than were physicians providing care as usual. However, clinician investment in training was required (two 1-hour training webinars) and patient encounters were lengthened by 28 minutes, suggesting challenges to scalability. The 10 Keys–APPRISE approach, in contrast, is designed to educate people directly about prevention outside the clinical encounter.

Our assessment of the 10 Keys–APPRISE program is limited by the absence of a comparison group and limited measures. Generalizability of these findings may also be limited by lack of diversity (eg, sex, race/ethnicity) among 10 Keys–APPRISE participants. However, the 10 Keys–APPRISE effort may address a gap in the prevention armamentarium. It directly educates older people about prevention outside the clinical encounter (unlike MOHR) and provides tools for incorporating prevention behaviors into daily life apart from management of existing conditions (unlike CDSMP). The link to Medicare preventive services strongly connects knowledge of risk factors to use of clinical preventive services. A key challenge going forward will be to determine whether participants in the program make better use of preventive services and whether these services lower the risk of incident chronic conditions.

## Appendix Text of 10 Keys to Healthy Aging Exam, University of Pittsburgh, Graduate School of Public Health, The Center for Aging and Population Health, a Centers for Disease Control and Prevention Prevention Research Center

Choose one: Male Female

Age (in years):

Choose one: Caucasian African American Hispanic Asian Other

1) What is the minimum number of hours of regular physical activity an older adult should get per week? [Circle one]

1. 6 hours

2. 1 hours

3. 5 hours

4. 2½ hours

[Answer: 2½ hours]

2) What is a normal blood pressure level? [Circle one]

1. 160/90 mm Hg

2. 120/80 mm Hg

3. 150/85 mm Hg

4. 142/72 mm Hg

[Answer: 120/80 mm Hg]

3) What is the preferred LDL cholesterol level? [Circle one]

1. Less than 130 mg/dL

2. Less than 200 mg/dL

3. Less than 100 mg/dL

4. Less than 150 mg/Dl

[Answer: Less than 100 mg/Dl]

4) How much calcium does someone age 50 and older need each day? [Circle one]

1. 800-1,000 mg

2. 1,200–1,500 mg

3. 1,500–1,800 mg

[Answer: 1,200–1,500 mg]

5) Every ___ year(s), all adults 50+ should have a colonoscopy unless they are at higher risk. [Fill in the blank] [Answer: 10 years]

6) Every ___ or __ year(s), women aged 50-74 in the general population should have a mammogram. [Fill in the blank] [Answer: 1 or 2 years]

7) Smoking is an addiction. True or False Circle One. [Answer: True]

8) What is the optimal fasting blood glucose level for older adults, with or without diabetes? [Circle one]

1. Less than 100 mg/dL

2. Less than 90 mg/dL

3. Less than 120 mg/dl

4. Less than 150 mg/Dl

[Answer: Less than 100 mg/dL]

9) Who (among people >65 years old) should receive a pneumonia immunization? [Circle one]

1. Healthy people

2. Smokers

3. Cancer patients

4. All of the above

[Answer: All of the above]

10) Who should receive an annual flu shot? [Choose all that apply]

1. Adults over age 50

2. Anyone with a chronic disease or weak immune system

3. Children 6 months and older

4. All of the above

[Answer: All of the above]

11) List 2 ways someone could maintain social contact. [Not scored]

1. Eg, Volunteer; join health club, book club, bicycle group, other group

2. Eg, Join organizations; travel with others

[Answer: both 1 and 2]

12) Depression is a normal part of aging. True or False [Circle one] [Answer: false]

13) People with osteoarthritis should avoid all physical activity in order to protect their joints. True or False [Circle One] [Answer: false]

14) Both men and women need to be concerned about bone loss as they age.

True or False [Circle One] [Answer: true]
